# Does sibling family structure matter in the emotion understanding development in preschoolers?

**DOI:** 10.3389/fpsyg.2024.1428087

**Published:** 2024-11-27

**Authors:** Margarita Aslanova, Margarita Gavrilova, Elena Iurina

**Affiliations:** ^1^Faculty of Psychology, Lomonosov Moscow State University, Moscow, Russia; ^2^Laboratory of Childhood Psychology and Digital Socialization, Federal Scientific Center for Psychological and Interdisciplinary Research, Moscow, Russia

**Keywords:** emotion development, emotion understanding, emotion comprehension, sibling position, preschoolers

## Abstract

The objective of the study was to investigate the impact of sibling family structure —including the number of children, age gaps, presence of a twin, sibling position, and gender composition — on emotion understanding and its development in children aged 5–6 years. A total of 409 preschoolers participated. Emotion understanding was assessed using The Test of Emotion Comprehension at the baseline and then again at a 1-year follow-up. In addition to the primary variables, executive functions (comprising visual and verbal working memory and cognitive flexibility) and non-verbal intelligence were controlled for in the analysis. We used the Dimensional Change Card Sort task to assess cognitive flexibility, the Sentence Repetition and Memory for Designs subtests of NEPSY-II to measure verbal and visual working memory, respectively, and Raven’s Coloured Progressive Matrices to assess non-verbal intelligence. Sibling data were obtained from the parent surveys, while psychological assessments were administered to children by psychologists. While sibling family structure influences emotion understanding, it does not significantly affect its development over a year. A more advanced reflective emotional understanding is associated with higher cognitive flexibility and having a younger sibling, compared to other sibling positions. The results of this study offer additional knowledge for parents, educators, family therapists, and child psychologists seeking a deeper understanding of emotional development in children. These professionals can design interventions and programs that leverage sibling and peer relationships to foster emotional development, encourage collaboration through age-diverse activities, and promote caregiving roles to enhance family and group dynamics.

## Highlights

- Performance of emotional understanding in 5-year-old children differs based on their sibling family structure.
- However, it is not associated with its development over a year.
- The presence of a younger sibling and a high level of cognitive flexibility are associated with more accurate reflective emotional understanding in preschoolers.
- Other sibling variables, such as the number of children in a family, age differences between them, the presence of a twin, and sibling gender composition, are not associated with preschoolers’ emotional understanding and its 1-year development according to the results of the study.

## Introduction

Emotional development plays a crucial role in children’s psychological growth, encompassing their ability to perceive, recognize, understand, and manage emotions. This capacity significantly impacts their overall well-being, social adaptation, and learning abilities (Roazzi et al., 2013). One crucial aspect of emotional development is emotion understanding (EU), which encompasses a range of emotional skills, including recognizing, labeling, and interpreting emotions in oneself and others. Emotion Comprehension (EC), as a subset of EU, refers specifically to understanding the causes and consequences of emotions in various contexts (Pons et al., 2002; Harris, 2016). While EU is a broader construct that includes several emotional competencies, EC focuses more narrowly on how emotions arise in response to external events, beliefs, and moral judgments. Thus, EC can be viewed as a more specific, cognitive aspect of EU that involves making sense of emotional reactions and their underlying reasons.

Emotional development in children is a complex multifactorial process influenced by both biological and genetic factors, as well as environmental factors, particularly the family context (Buss et al., 2019; Israel et al., 2015). While researches indicate that the family environment, especially parenting styles, significantly influences a child’s emotional development (Morris et al., 2007; Al-Elaimat et al., 2020), the specific impact of sibling family structure on EU remains relatively unexplored.

### EU and siblings within the context of the family environment

According to the theory of family systems (Minuchin, 1974), sibling relationships are essential elements, or substructures, within the broader family framework. This perspective posits that families are intricate, multifaceted, and ever-changing entities comprising various interrelated subcomponents, such as marital, parent–child, grandparent-child, and sibling subsystems. While parents are often regarded as the designers of the family, setting rules and boundaries that define relationships across generations, siblings are seen as key players who are integral to numerous family functions. In the review (Kramer, 2014), the author highlighted the role of siblings’ relationships with each other and other family members in their cognitive, social, and emotional adaptation. These effects can be both direct, arising from the interactions between siblings, or indirect, stemming from a child’s influence on their parents’ caregiving behaviors towards other siblings (Brody et al., 2003). Additionally, the concept of differential treatment by parents represents another avenue through which having a sibling can impact a child’s psychological development (Kowal et al., 2002). Sibling relationships play a crucial role in children’s social, emotional, moral, and cognitive development (Stormshak et al., 2009; Whiteman et al., 2011; Howe et al., 2022). Children who receive emotional support from their older siblings (in the form of care, acceptance, and boosting of self-esteem) during interparental conflict exhibit fewer behavioral or emotional issues compared to children whose older siblings are less supportive (Jenkins, 2013). Sibling relationships can also have adverse effects on children’s development. Younger siblings who are raised with aggressive older siblings face significant risks of developing conduct problems, underperforming in school, and experiencing limited positive interactions with their peers (Bank et al., 1996). Understanding the influence of sibling family structure on emotional development gains particular importance when examining preschoolers’ development, as this sensitive period plays a critical role in shaping their EU and lays the foundation for future emotional competence and social interactions (Goleman, 2020).

The developmental model behind the study is based on ecological systems theory (Bronfenbrenner, 1979), which posits that a child’s development is influenced by multiple layers of their environment, with the family (microsystem) being one of the most critical. In this context, the sibling family structure represents an essential aspect of the children’s immediate environment, which may be associated with their emotional development.

The study adopts an ecological approach by focusing on how the sibling family structure, as part of the child’s social context, may contribute to emotional development. By examining structural variables, the study explores how they may provide a developmental environment that influences emotional growth.

### Current study

The current study focuses on analyzing the EU, a crucial aspect of emotional development, during the formative preschool years. We hypothesize that variations in sibling structure, such as the number of children in a family, age differences with them, the presence of a twin, sibling position, and sibling gender composition, may be associated with preschoolers’ EU and its development. By examining the potential associations between sibling family structure and EU at a specific time point (age of 5 years, T1) and its developmental progression over a year (5–6 years, T2-T1), the study aimed to gain insights into the role of siblings in shaping EU during the preschool period.

In this study, “sibling family structure” refers to a defined set of characteristics: the number of siblings, their gender, age, and the age gap between them and the participant. These structural variables were chosen for their simplicity, objectivity, and consistency in measurement, enabling reliable comparisons and ensuring reproducibility. Since our participants are five-year-old children, sibling relationships and interactions at this stage are generally more straightforward, with fewer entrenched dynamics, making the inclusion of complex interpersonal variables unnecessary for the study’s aims.

While other studies have focused on relational factors, such as the quality of sibling bonds and daily interactions, our approach emphasizes replicable, universal patterns. This focus establishes a framework for future research to explore more intricate aspects of sibling relationships in emotional development.

In the context of Theory of Mind (ToM), siblings are likely to play a role in a child’s understanding of others’ emotions and mental states. Although the study does not specifically investigate sibling interactions, it suggests that the presence of siblings creates contextual opportunities for emotional and social cognition. These everyday sibling interactions may foster early signs of understanding others’ minds and emotions, even if this understanding develops more fully and is formally assessed later.

Our study uniquely examines how sibling family structure may affect EU and its development in preschool children, an area not well-explored. Unlike most research that focuses on parents or sibling relationships, we analyze demographic factors such as sibling number, age gaps, presence of twins, sibling position, and gender composition. This approach offers valuable insights into how these factors might influence emotional development during critical preschool years, setting our study apart with a fresh perspective on children’s emotional growth and development.

In studying the EU in children and its association with sibling family structure, it becomes pertinent to consider underlying cognitive structures that have previously been linked to the development of the EU. The importance of these factors is emphasized by a plethora of research that suggests their foundational roles in the development of emotion recognition.

### Cognitive factors and EU: control variables justification

Non-verbal intelligence, verbal and visual working memory, and cognitive flexibility were included as control variables, given their associations with EU in previous studies (Morra et al., 2011; Ye et al., 2018; Wang et al., 2021; Veraksa et al., 2022; Chichinina and Gavrilova, 2022; Zakharova and Machinskaya, 2023).

A salient characteristic that has been frequently associated with EU in children is *non-verbal IQ*. Multiple studies underscore the relationship between IQ and the comprehension of emotions (Bennett et al., 2005; Pears and Fisher, 2005; Sullivan et al., 2008). In particular, non-verbal intelligence has been highlighted as a significant predictor of various essential aspects of emotion understanding (Albanese et al., 2010). The cognitive processes foundational to analytical intelligence, including the ability to address novelty and reason with new information, are instrumental in aiding children to discern and interpret pertinent emotional cues (Albanese et al., 2010).

Additionally, *cognitive flexibility* is an important determinant in the development of the EU. This is underpinned by research indicating that the acquisition of EU in children requires inherent cognitive flexibility, which empowers them to re-evaluate their initial perceptions and adjust their behavior contextually (Silkenbeumer et al., 2016). Such a relationship is further bolstered by findings that link heightened cognitive flexibility to enhanced EU, especially in terms of recognizing and responding to both self and external emotional states (Wang et al., 2021). This observation aligns with theoretical models that suggest a deep connection between the evolution of the ToM and cognitive flexibility (Jacques and Zelazo, 2005; Farrant et al., 2012).

*Working memory*, which pertains to the concurrent maintenance and processing of information (Baddeley, 1986; Baddeley and Hitch, 2000), emerges as a robust predictor of developmental variances in emotion comprehension (Morra et al., 2011) and emotional competence in general (Pons et al., 2002; Mutter et al., 2006; Baddeley, 2012; Diamond, 2013).

In light of the above, it’s clear that non-verbal intelligence, cognitive flexibility, and components of executive functions, particularly working memory, are vital controls in studying EU in children. Incorporating these control variables ensures a comprehensive analysis that accounts for previously established cognitive predictors of the EU.

The research may have practical implications for parents, educators, and child psychologists in better understanding children’s emotional development, which is an essential factor in their overall psychological well-being.

## Methods

### Participants and procedure

The current study took place in kindergartens in Moscow, from March, 2022 to March, 2024. The study was reviewed and approved by the Ethics Committee of Russian Psychological Society Ethics Committee. Written informed consent to participate in this study was provided by the participant’s legal guardian/next of kin. The exclusion criterion was based on the parent’s or legal guardian’s refusal to sign the child’s participation agreement. The sample consists of 409 five-year-old typically developing preschool children, of which 47.2% (N = 193) were boys. Sampling in the study was based on a probabilistic sampling methodology. We used stratified random sampling, where kindergartens were selected based on a range of socioeconomic and demographic factors to ensure a representative sample of preschool children in Moscow. This method helps reduce selection bias and increase the generalizability of the findings.

Sibling data were collected by surveying parents. Parents responded to online questionnaires where they were asked to provide information on the number of children in the family, their gender, and age. Participants were subject to psychological assessment by all tests at the baseline, and the Test of Emotion Comprehension was held twice, with a one-year break. Qualified child psychologists conducted the assessments in a quiet room at the kindergartens during the first half of the day. The order of application of tests was counterbalanced. The assessment consisted of three sessions, each lasting 20 min.

### Measures

Russian version of the *Test of Emotion Comprehension (TEC)* (Pons and Harris, 2000; Veraksa et al., 2021) applied the assessment of the children’s ability to understand emotions.

The Test of Emotion Comprehension (TEC) is a well-established tool for assessing children’s EU (Pons and Harris, 2000). It evaluates children’s ability to recognize emotions, understand emotional causes, and consider how beliefs and moral judgments shape emotional reactions. The TEC provides a nuanced picture of emotional development across different levels—external (emotion identification), mental (understanding of beliefs and desires), and reflective (understanding mixed emotions and moral consequences). The psychometric properties of TEC have been validated across various cultural contexts, demonstrating its reliability and validity (Albanese et al., 2010). However, limitations of TEC include its reliance on verbal explanations of emotional scenarios, which may be influenced by a child’s language abilities. A comprehensive description of these strengths and weaknesses will be added.

The study employed the Russian adaptation of the TEC, validated for use with preschool-aged children (Veraksa et al., 2021). Boys and girls were given the appropriate versions of the test, as specified in the original TEC format.

The TEC is designed for children aged between 3 to 11 years and provides versions for both boys and girls. The Test uses a sequence of illustrated stories to evaluate various facets of emotional comprehension. Each page presents four potential emotional outcomes depicted through facial expressions (the five options are: happy, sad, angry, afraid, and neutral). The children’s task is to match the emotional facial expression to the described situation. The method evaluates: (1) identifying emotions via facial expressions, (2) comprehension of emotions triggered by external events, (3) acknowledging desires as a cause of emotions, (4) the influence of beliefs in shaping emotions, (5) the role of memory in evaluating emotional states, (6) the ability to control emotions, (7) the capability to mask or hide emotions, (8) recognizing that a person can experience mixed emotions (for instance, fear and happiness simultaneously) in a given circumstance, and (9) understanding the impact of moral values on emotions. The test provides an opportunity to evaluate three different levels of emotion comprehension: external, mental, reflective, and total score. The external level allows assessment of a child’s ability to understand external reasons for emotional reactions and comprises three sections: recognition, external cause, and desire. The mental one includes various psychological aspects of these reactions and encompasses the subsequent three sections: belief, reminder, and regulation. The reflective level is related to a child’s understanding of how he or she can track and analyze one’s own emotional states and includes three sections: hiding, mixed, and morality. Each component is classified as either pass or fail depending if the reply was correct or not. Each level score, ranging from 0 to 3, is the cumulative sum of the scores attained in each associated component. Therefore, the total score for emotional comprehension could vary from 0 to 9 points.

*The Dimensional Change Card Sort task (DCCS)* (Zelazo, 2006) was applied for the assessment of cognitive flexibility. During the DCCS task, a child is instructed to categorize cards across three stages, each governed by different rules. Initially, the categorization is dependent on the color of the images on the cards (pre-switch trial). Then it switches to be based on the shape (switch trial), and finally, on conflicting rules where the sorting depends on the presence of a frame on the card which dictates whether the card should be sorted by color or shape (post-switch trial). The total score ranges from 0 to 24.

*The Sentence Repetition subtest* of *NEPSY-II* is designed to assess verbal working memory and measures a child’s ability to hold, process, and reproduce verbal information (Korkman et al., 2007). During the task, a child must repeat the sentence that the psychologist read aloud. The test comprises 17 sentences that gradually increase in their complexity in terms of length and syntactic structure. Any word omissions, substitutions, or additions were counted as errors, as were any alterations to the word order. The test was ended if a child scored 0 points for four consecutive sentences. The final score can range from 0 to 30.

*The Memory for Designs subtest* of *NEPSY-II* (Korkman et al., 2007) was used to evaluate visual working memory. This test measures two components of visual memory— the recall of content and the spatial positioning of images. Each task earned two points for every accurately chosen card (“Content score”) and one for every accurately identified location (“Spatial score”). Two additional points were awarded on each trial if a child correctly identified a card and positioned it in the right place (“Bonus score”). The total score ranges from 0 to 100 points.

*The Raven’s Coloured Progressive Matrices* (Raven, 1998) is a commonly employed task for evaluating visuo-spatial pattern recognition, often viewed as an assessment of non-verbal intellect and abstract thought. To effectively complete this challenge, participants must choose one out of six potential pieces which accurately fills a missing space in a visual design. Participants can score between 0 and 36 points on the Raven’s Coloured Progressive Matrices test.

### Data analysis

The distributions of all variables were examined using the Kolmogorov–Smirnov test, and since all numeric variables exhibited a non-normal distribution, non-parametric tests were applied for further analysis. Median and Interquartile Range (IQR) were used as the most informative characteristics of our quantitative variables, while Mean and Standard Deviation (SD) were also provided for the broader context of the data.

Descriptive statistics were performed for all variables. Spearman correlation analysis was used to investigate the association between numeric sibling variable reflected age differences and EU at baseline.

The Mann–Whitney U test was applied in the analysis for EU and sibling variables with two groups, such as the presence of a sibling, the presence of a twin, and having all siblings the same gender as a participant; while for variables with three or more groups, such as a number of children in a family, sibling gender structure, and sibling position, was used the Kruskal-Wallis test with *post hoc* analysis using Dunn’s test with Bonferroni correction.

To determine TEC differences at the 1-year follow-up, the Wilcoxon signed-rank test was performed. Spearman correlation analysis, the Mann–Whitney U test, and the Kruskal-Wallis test were used to determine the strength and direction of the association between the aforementioned sibling variables and TEC development in 1 year. The TEC development variables (dif. TEC) are presented by the difference between two point measures from second to first (T2-T1).

We implemented an ordinal logistic regression analysis with the dependent variables that showed significant correlations with sibling variables in the bivariate analyses, by controlling for Non-verbal Intelligence (Raven), Cognitive Flexibility (DCCS), Verbal Working Memory (Sentences Repetition), and Visual Working Memory (Memory for Designs).

Statistical analysis was performed using Version 22 of SPSS for Windows, and differences were considered significant at *p* < 0.05.

## Results

### Descriptive analysis of all study variables at baseline

A descriptive analysis of data obtained by psychological assessment is shown in Table 1 and sibling variables are presented in Table 2.

**Table 1:** Control variables data.

Variable	Median/IQR	Mean (SD)/Range in our sample	N/missed
Non-verbal intelligence	12.00/12	13.15 (6.92) 0–32	408/1
Cognitive flexibility	18.00/4	18.77 (2.89) Oct-24	409/0
Verbal working memory	18.00/7	18.35 (4.90) Feb-38	408/1
Visual working memory	37.00/8	37.91 (5.46) 22–48	407/2

**Table 2:** Sibling family structure data.

Variable	Median/IQR Mean (SD)	% (N)	N/Missed
Has siblings:			409/0
yes		50.4% (206)	
no		49.6% (203)	
Quantity of siblings (numeric):	1.00/1		409/0
0		49.6% (203)	
1		35.9% (147)	
2		11.7% (48)	
3		2.2% (9)	
4		0.5% (2)	
Number of children in the family (grouped):			409/0
one child		49.6% (203)	
two children		35.9% (147)	
three and more children		14.4% (59)	
Birth order (if a participant has siblings):			206/0
1	2.00/1	41.7% (86)	
2		44.7% (92)	
3		12.1% (25)	
4		0.5% (1)	
5		1% (2)	
Gender of siblings:			206/0
all brothers		45.1% (93)	
all sisters		37.9% (78)	
mixed		17% (35)	
a child has the same gender as siblings		38.3% (79)	
Minimal age difference (years)	3.00/5 4.83(4.49)		183/23
Maximum age difference (years)	4.00/7 6.01(5.16)		183/23
Has a twin*		5.1% (21)	409/0
Sibling position (wide variable):			385/24
Singleton		49.6% (203)	
Eldest		11.2% (46)	
Eldest twins		0.7% (3)	
Middle		3.7% (15)	
Middle twins		0.2% (1)	
Youngest		24.4% (100)	
Youngest twins		1% (4)	
Sibling position**:			377/32
Singleton		49.6% (203)	
Eldest		11.2% (46)	
Middle		3.7% (15)	
Youngest		24.4% (100)	
Twins only*		3.2% (13)	

* The “Twins only” variable includes twins without any other siblings, while the “has a twin” group includes both twins only and twins with siblings. ** Eldest, middle, and youngest sibling position variables include eldest twins, middle twins, and youngest twins, respectively. The wide variable of Sibling position is presented for reference, while Sibling position was used for statistical analysis.

### Association of EU and sibling variables at baseline

The association of EU and sibling variables at baseline is presented in Table 3. The TEC Reflective score was significantly higher in the eldest children group compared to the group of singleton children (H (3) =11.180; *p* = 0.011*). No significant differences were observed among other sibling positions for this variable. Other EU variables did not exhibit statistically significant associations with sibling variables at the baseline.

**Table 3:** Association of EU and its 1-year development with sibling variables.

	Sibling variables							
Emotion understanding variables	Presence of siblings (yes/no)	Number of children in the family (1,2,3+)	Same-Gender siblings (yes/no)	Gender of siblings (sis/bro/mix)	Minimal age difference	Maximum age difference	Has a twin (yes/no)	Sibling position (singleton, eldest, middle, youngest, twins only)
External	U = 20416.5 *p* = 0.845	H(2) = 1.284 *p* = 0.526	U = 4920.0 *p* = 0.920	H(2) = 0.387 *p* = 0.824	r = 0.028 *p* = 0.706	r = 0.053 *p* = 0.481	U = 3575.0 *p* = 0.490	H(4) = 1.863 *p* = 0.761
Mental	U = 19639.0 *p* = 0.383	H(2) = 1.248 *p* = 0.536	U = 4441.0 *p* = 0.184	H(2) = 1.301 *p* = 0.522	r = 0.075 *p* = 0.314	r = 0.036 *p* = 0.633	U = 3808.5 *p* = 0.914	H(4) = 1.735 *p* = 0.784
Reflective	U = 18944.0 *p* = 0.137	H(2) = 2.323 *p* = 0.313	U = 4847.5 *p* = 0.787	H(2) = 0.781 *p* = 0.677	r = 0.001 *p* = 0.987	r = 0.018 *p* = 0.807	U = 3795.5 *p* = 0.894	**H(4) = 12.151** *p* = 0.016*
Emotion	U = 20310.0 *p* = 0.800	H(2) = 0.335 *p* = 0.846	U = 4505.5 *p* = 0.267	H(2) = 1.337 *p* = 0.512	r = 0.042 *p* = 0.573	r = 0.042 *p* = 0.574	U = 3628.5 *p* = 0.644	H(4) = 4.832 *p* = 0.305
Dif. External	**U = 12,081** p = 0.046*	H(2) = 3.996 *p* = 0.136	U = 4809.5 *p* = 0.852	H(2) = 0.053 *p* = 0.974	r = −0.102 *p* = 0.171	r = −0.102 *p* = 0.173	U = 3062.0 *p* = 0.769	H(4) = 8.104 *p* = 0.088
Dif. Mental	U = 13477.5 *p* = 0.882	H(2) = 3.469 *p* = 0.177	U = 4235.5 *p* = 0.101	H(2) = 0.693 *p* = 0.707	r = −0.080 *p* = 0.282	r = 0.018 *p* = 0.806	U = 2818.5 p = 0.383	H(4) = 1.421 *p* = 0.841
Dif. Reflective	U = 12985.5 *p* = 0.467	H(2) = 0.583 *p* = 0.747	U = 4597.5 *p* = 0.479	H(2) = 1.397 *p* = 0.497	r = −0.065 *p* = 0.387	r = −0.108 *p* = 0.148	U = 3158.0 *p* = 0.977	H(4) = 4.053 *p* = 0.399
Dif. Emotion	U = 12801.5 *p* = 0.353	H(2) = 1.680 *p* = 0.432	U = 4738.0 *p* = 0.733	H(2) = 0.008 *p* = 0.996	r = −0.098 *p* = 0.188	r = −0.071 *p* = 0.340	U = 3054.5 *p* = 0.781	H(4) = 3.098 *p* = 0.542

The eldest children group exhibits higher TEC reflective values than the singleton children group. Youngest, middle, and children, who have only twin siblings, do not exhibit statistically significant differences in this factor either with the aforementioned groups of children or with each other. Children without siblings show a higher increase in TEC External variable in 1-year follow-up compared to those with siblings. Bold values indicate statistically significant differences (*p* < 0.05) based on the applied statistical tests.

Ordinal regression analysis revealed that the independent variable sibling position and control variable cognitive flexibility significantly influence TEC Reflective scores. Other control variables did not exhibit statistical significance in the regression model. By sequentially swapping the reference dummy variables, it was found that the eldest child and middle child groups perform statistically significantly better compared to the singleton, youngest child, and twin groups. Eldest children outperformed middle children. No differences were observed between singleton children, youngest children, and twins. In other words, the presence of a younger sibling significantly predicts better TEC Reflective scores. The significant coefficients of sibling position categories relative to various reference dummy variables are presented below.

Reference singleton: eldest (B = −0.946, SE = 0.303, Wald = 9.713, *p* = 0.002*, OR = 0.388, CI = −1.540 – −0.351), middle (B = −1.037, SE = 0.497, Wald = 4.351, *p* = 0.037*, OR = 0.355, CI = −2.012 – −0.063);

Reference youngest: eldest (B = −0.987, SE = 0.322, Wald = 9.404, *p* = 0.002*, OR = 0.373, CI = −1.619 – −0.356), middle (B = -1.080, SE = 0.509, Wald = 4.501, *p* = 0.034*, OR = 0.340, CI = −2.078 – −0.082);

Reference twins: eldest (B = −1.051, SE = 0.396, Wald = 7.060, *p* = 0.008*, OR = 0.350, CI = −1.826 – −0.276), middle (B = −1.144, SE = 0.559, Wald = 4.191, *p* = 0.041*, OR = 0.319, CI = −2.238 – −0.049);

Reference middle child: eldest (B = −0.778, SE = 0.386, Wald = 4.054, *p* = 0.044*, OR = 0.459, CI = −1.535 – −0.021).

Cognitive flexibility score (B = 0.084, SE = 0.035, Wald = 5.758, *p* = 0.016*, OR = 1.087, 95% CI =0.015–0.153) was positively associated with TEC Reflective. The significant model (X^2(9) = 21,940, *p* = 0.009*) explained 5.8% of the variance (Nagelkerke R^2).

### Development of EU in 1-year follow-up and its association with sibling variables

EU scores in T1 and T2 time points and their differences in the 1-year follow-up are presented in Table 4. Wilcoxon Signed-Rank Test for paired samples revealed statistically significant increases in all three parameters of EU from T1 to T2 in our sample.

**Table 4:** EU scores in T1 and T2 time points and its differences in 1-year follow-up.

	T1: Median/IQR; Mean (SD)	T2: Median/IQR; Mean (SD)	T2-T1 differences; Median/IQR; Mean (SD) Z, *p*-value
External	3.00/1 2.63 (0.601)	3.00/0 2.84 (0.417)	0.00/1; 0.246 (0.741) Z = −5.710, *p* = 0.000*
Mental	1.00/1 1.27 (0.823)	2.00/1 1.47 (0.640)	0.00/1 0.20 (0.968) Z = −3.783, *p* = 0.000* 0.00/1
Reflective	1.00/2 1.08 (0.871)	1.00/1 1.47 (0.898)	0.362 (0.202) Z = −5.370, *p* = 0.000* 1.00/2
Emotion	5.00/2 4.98 (1.471)	6.00/2 5.78 (1.238)	0.813 (1.823) Z = −7.550, *p* = 0.000*

Participants/missed: T1: *N* = 406/3; T2: *N* = 339/70; T2-T1 Diff: *N* = 337/72. *The Wilcoxon Signed-Rank Test for paired samples revealed statistically significant increases in all four EU parameter scores—External, Mental, Reflective, and Emotion—from T1 to T2. Bold values indicate statistically significant differences (*p* < 0.05) based on the applied statistical tests.

Association of EU Differences T2-T1 and sibling variables are shown in Table 3: children without siblings show a higher increase in TEC External variable in the 1-year follow-up compared to those with siblings (U = 12,081; *p* = 0.046*). Although the unadjusted comparison showed a difference in the change in TEC External scores between children with and without siblings, this difference was not statistically significant when other variables were controlled in the regression model. Specifically, once these variables were accounted for, the overall regression model failed to reach statistical significance. Furthermore, other EU development variables did not demonstrate any significant associations with sibling factors.

## Discussion

The present study aimed to determine the possible association of sibling family structure and EU, and also its development in children aged 5–6 years old. Our key findings indicate that sibling family structure is associated with the EU, but not with its dynamics in a 1-year follow-up. Children who have a younger sibling compared to other sibling positions demonstrate better reflective emotional understanding. The association was confirmed by the regression analysis under the adjustment of control variables, which additionally demonstrated the impact of cognitive flexibility in this level of EU. Other sibling variables, such as the number of children in a family, age differences with them, the presence of a twin, and sibling gender composition, were not show association with preschoolers’ EU and its developmental trajectory according to our results. Other control variables also did not show significance in the regression model.

The reflective level of the EU is the most complex of other levels and evaluates different components: emotion regulation, understood as the management of negative emotions through cognitive strategies; mixed emotions, reflecting the understanding that people can experience conflicting mixed emotions in certain situations; and moral emotions, which show awareness that negative emotions arise from morally unacceptable behavior, and positive emotions stem from morally commendable actions.

Regarding the concept of the ToM, which is closely related to the EU, we can reference the study by Paine et al. (2018), whose findings align with the results of our current research. The authors found that younger siblings can foster the development of ToM beyond the preschool years. Having a younger sibling often necessitates taking on some level of a caregiving or leadership role, which can foster a deeper understanding of others’ needs and emotions.

Older siblings can convey cognitive concepts and language skills to their younger siblings by adapting their teaching methods to suit the developmental stage of the learner. This adaptability in instructional behavior is enhanced by the development of perspective-taking skills, allowing for a nuanced comprehension of others’ cognitive states (Maynard, 2002). Older siblings who take on the responsibilities of teaching and caregiving experience improved reading and language achievement scores. They also develop a stronger sense of competence in their caregiving role and acquire the skill of balancing their own concerns with the needs of others more rapidly compared to older siblings who do not take on these roles with their younger siblings (Zukow-Goldring, 1995). The experience of relating to a younger sibling helps children learn about the desires, needs, and ideas of other individuals as separate from their own while acquiring the language of emotion (Dunn, 1988; Brown and Dunn, 1992).

Talking about emotion regulation as a component of EU in older siblings we can refer to the study of Miller et al. (2000) who highlighted that older children were better than their toddler-age siblings at regulating jealousy responses and engaging in focused play and showed greater behavioral consistency across parents compared to his younger sibling, indicating internalization of emotion regulation style. Older siblings possess better emotion regulation strategies and are better able to maintain their focus more easily under challenging conditions (Kopp, 1992).

High cognitive flexibility is also associated with better performance in reflective EU according to the results of the study. In essence, having a younger sibling provides the practical field for applying cognitive skills, while cognitive flexibility enriches these interactions, making them more meaningful and educational. Both factors, in tandem, contribute to an enhanced understanding of emotions in preschoolers.

Children without siblings show a higher increase in TEC External variable in 1-year follow-up compared to those with siblings. There could be several reasons why children without siblings show a greater increase in understanding external emotional cues. Only children might have more opportunities to interact with adults and might therefore be exposed to more complex emotional interactions. Additionally, without siblings, only children may also rely more heavily on their peer interactions to understand external emotional cues. This reliance might drive a more significant increase in this skill over time.

Although the unadjusted comparison showed a difference in the change in TEC External scores between children with and without siblings, this difference was not statistically significant when other variables were controlled in the regression model. Specifically, once these variables were accounted for, the overall regression model failed to reach statistical significance. This suggests that other factors, which might include parental involvement, socioeconomic status, personality traits, and mental and physical health could be more influential in driving changes in TEC External scores over time (Albanese et al., 2010; Morra et al., 2011; Wang et al., 2021).

Based on the results of the study, having a younger sibling may positively impact the EU in children. This could possibly be heightened by feelings of responsibility, the need to teach younger siblings skills and games, and the desire to approach the “adult” role within the family. Educators are encouraged to engage children of different age groups in joint activities and promote mentoring by older children for younger ones.

## Limitations and future directions

The limitation of the study is the absence of a variable assessing the quality of sibling relationships. For instance, the authors (Brody, 1998; Stormshak et al., 2009; Whiteman et al., 2011; Kramer, 2014; Howe et al., 2022) pointed out the link between the quality of sibling relationships and emotional development in children. Additionally, EU was assessed in our study by only one tool: the Test of Emotion Comprehension. Incorporating alternative metrics for measuring EU could enrich the existing body of research. Given that our research was limited to a one-year follow-up, extending the study to examine the long-term impact of sibling family dynamics on EU would be beneficial. Also, our study focuses on the preschool developmental period. Investigating how sibling family structure might influence EU at other developmental stages also would be informative. Future studies could adopt a more dynamical and ecological approach by incorporating longitudinal designs and qualitative measures. For example, investigating how the quality of sibling interactions (e.g., caregiving, cooperation, or rivalry) evolves over time and its effect on emotional understanding (EU) would provide a richer and more holistic view of the development process. This could involve using qualitative methods, such as observations or parent–child interviews, to capture the emotional dynamics within sibling interactions. Additionally, long-term studies examining how the influence of sibling relationships changes at different stages of development—such as middle childhood or adolescence—would provide further insight. Moreover, adopting an ecological systems framework (Bronfenbrenner, 1979) would enhance our understanding of how different layers of a child’s environment (family, school, peers) interact to shape emotional development. For example, future research could investigate how parental involvement, socioeconomic factors, or even peer relationships mediate or moderate the effect of sibling dynamics on EU.

## Conclusion

Performance of emotional understanding in 5-year-old children differs based on their sibling family structure, but does not have an association with its dynamics in a 1-year follow-up: the presence of a younger sibling predicts a better reflective EU. Also, cognitive flexibility as a control variable had a positive influence on this level of EU. Other sibling variables, such as the number of children in a family, age differences with them, the presence of a twin, and sibling gender composition, were not associated with preschoolers’ EU and its developmental trajectory according to our results.

The results of this study offer additional knowledge for parents, educators, and child psychologists seeking a deeper understanding of emotional development in children. Based on these findings, which align with the concept of age-diverse development, educators might consider involving children from various age groups in collaborative activities and promoting mentorship by older children. These results serve as a useful reference point for specialists seeking to integrate sibling relationships into developmental support, particularly in cases involving unique developmental traits, thereby benefiting both individual and family dynamics. For example, the finding that the presence of a younger sibling is linked to more accurate reflective emotional understanding can guide family-based interventions. Although the study focused on structural elements rather than the quality of sibling interactions, practitioners can still encourage family environments where older siblings are engaged in caregiving roles or cooperative tasks with younger siblings. This can be promoted through structured family activities that support interaction between siblings, to leverage the existing sibling structure to enhance emotional competence in both older and younger children.

## Data Availability

The raw data supporting the conclusions of this article will be made available by the authors, without undue reservation.
